# Characterization of the complete chloroplast genome of *Barbella flagellifera* (Cardot) Nog. 1938 (Bryidae, Meteoriaceae)

**DOI:** 10.1080/23802359.2024.2318393

**Published:** 2024-02-26

**Authors:** Zhen-Yan Yuan, Qiang Wang, Mamtimin Sulayman

**Affiliations:** a College of Ecology and Resources Engineering, Wuyi University, Wuyishan, PR China; b Fujian Provincial Key Laboratory of Eco-Industrial Green Technology, Wuyi University, Wuyishan, PR China; c Zhejiang Museum of Natural History, Hangzhou, PR China; d College of Life Science and Technology, Laboratory of Biological Resources and Genetic Engineering, Xinjiang University, Urumqi, PR China

**Keywords:** *Barbella flagellifera*, chloroplast genome, Meteoriaceae, phylogeny

## Abstract

Whether *Barbella flagellifera* (Cardot) Nog. belongs to *Neodicladiella* has not yet been determined, and a study of the complete chloroplast (cp) genome of *B. flagellifera* would aid in determining its evolutionary position. In this study, we aimed to sequence the complete cp genome of *B. flagellifera*, an epiphytic species found in tropical and subtropical forests. The complete cp genome is 125,025 base pairs (bp) long and appears as a typical quadrilateral structure with a large single-copy (86,854 bp), a small single-copy (18,473 bp), and a pair of inverted repeats (19,698 bp). The overall GC content of the sequence is 41.0% and the genome encodes 127 genes, including 82 protein-coding genes, 37 tRNAs, and eight rRNAs. Phylogenetic analysis reveals that *B. flagellifera* clustered into a clade with other Hypnales groups with high bootstrap support. The complete cp genome presented here will provide helpful information for species identification of *Barbella* genus and *Neodicladiella* genus in Meteoriaceae.

## Introduction

Meteroriaceae mainly comprise epiphytic mosses occurring in tropical and subtropical regions. It is easy to recognize them by their pendent habits, a characteristic earlier regarded important for the delimitation of this family (Naren and Jia 2020). *Barbella flagellifera* (Cardot) Nog. is reported by Meteoriaceae as an accepted name in the genus *Barbella* (Noguchi 1976). The *Neodicladiella* genus was established by Buck, who revised the taxonomy of Meteoriaceae and separated the group into a new genus including one species, *Neodicladiella pendula* (Sull.) (Buck 1994). Huttunen, excluding *Barbella flagellifera* (Cardot) Nog. from *Barbella* and placing it in *Neodicladiella*, renamed *Neodicladiella flagellifera* (Cardot) (Huttunen and Quandt 2007). To date, *N. flagellifera* is reported as a homotypic synonym of *B. flagellifera* (Tropicos 2023). The characters of *Barbella* are more similar to those of the genus *Neodicladiella*, such as well developed alar cells and 1–2 papillose leaf cells. Currently, there have not been any laboratories using the whole plastome of *B. flagellifera* for research for molecular biology identification. Consequently, we performed sequencing technology and phylogenetic analysis to publish the whole chloroplast (cp) genome of *B. flagellifera* and enhance the species delimitation in Meteoriaceae.

## Materials and methods

**Figure 1.**
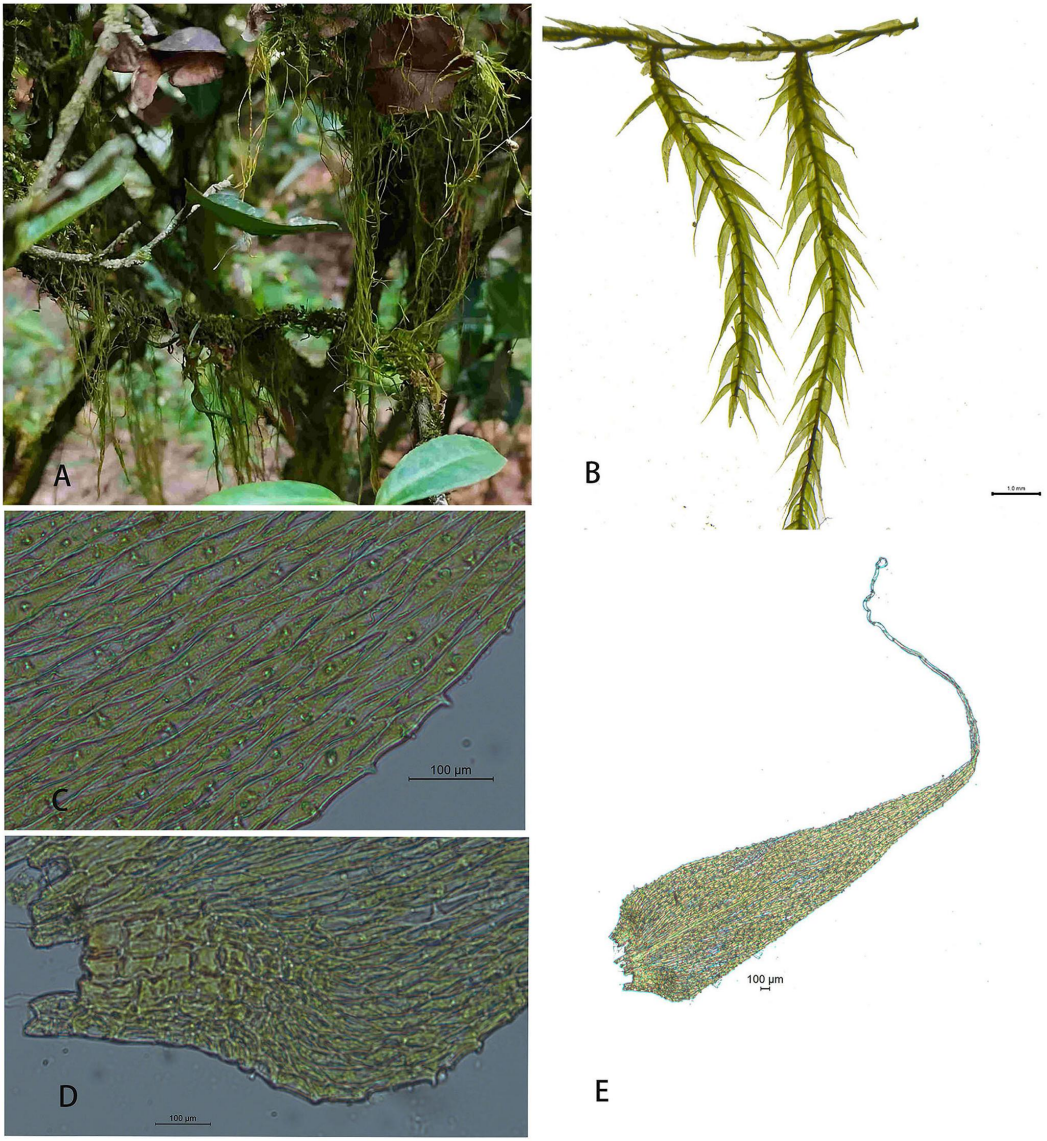
Photograph of *Barbella flagellifera* (Cardot) Nog. (photographed by Zhenyan Yuan in Wuyishan, China). Plants slender, primary stems elongate, secondary stems elongate, delicate, and pendulous. Branch leaves appressed, narrow-triangular, leaf bases constricted, each gradually narrowing to a long piliferous apex, usually flexuose. Leaf cells unipapillose. (A and B) Plant. (C) Median cells of branch leaf. (D) Alar cells. (E) Branch leaf.

Plant samples of *B. flagellifera* were collected from the Wuyi Mountains (117.929444°E, 27.385833°N), Fujian Province, China, and deposited at the Herbarium of Wuyi University under voucher number WY17 (Figure 1) (www.wuyiu.edu.cn, Yuan Zhen-Yan, 65482398@qq.com). Fresh samples were sent to Nanjing Genepioneer Biotechnology Co., Ltd. (Nanjing, China) for DNA extraction using a plant genomic DNA kit (Tiangen Biotech, Beijing, China) and for DNA library construction. The library was sequenced using an Illumina NovaSeq 6000 sequencing platform (Illumina, San Diego, CA). The cp genome was assembled using SPAdes 3.10.1 (Bankevich et al. 2012). Two approaches were used to annotate the cp genome; prodigal 2.6.3, hmmer3.1, and aragorn 1.2.38 were used to predict the CDS, rRNA, and tRNA, respectively. The cis-splicing gene maps were carried out by cpgview (https://doi.org/10.1111/1755-0998.13729). The other approach used the *Haplocladium microphyllum* genome (GenBank accession number: NC_050974.1) as a reference for annotation using BLAST 2.6. Gene annotation was performed using manual boundary corrections. The cp genome information, under an annotation number, was submitted to the NCBI GenBank database (NCBI accession number: OQ934045). A circular cp genome map was constructed using the OGDRAW program (Tillich et al. 2017), obtaining a more accurate cp genome. The minimal, maximal, and average read mapping depths for assembled genomes were ×8, ×1060, and ×685.66 (Figure S1), respectively. The cis and trans-splicing map of *B. flagellifera* was drawn through CPGview (Liu et al. 2023). To identify the phylogenetic position of *B. flagellifera*, a maximum-likelihood (ML) tree was reconstructed based on the complete cp genomes of 29 species using IQTREE2 (Minh et al. 2020).

## Results and discussion

**Figure 2.**
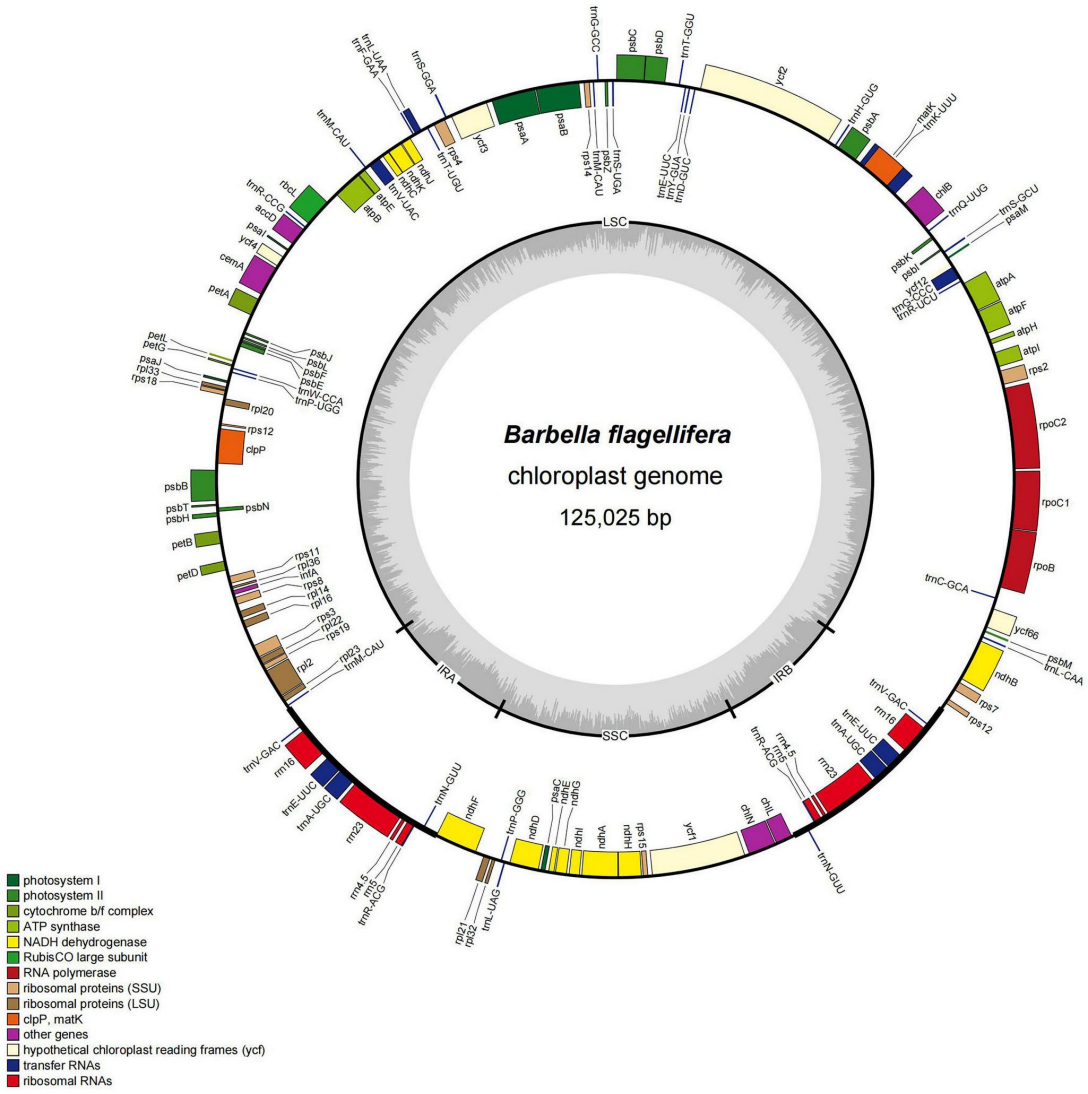
Map of the chloroplast genome *Barbella flagellifera* (Cardot) Nog. The boxes of different sizes and colors on the outermost circle represent genes and their length. The inner and outer boxes of the outermost circle represent genes transcribed clockwise and counter-clockwise. The darker gray area in the inner circle represents the changes in GC content at different positions, whereas the lighter gray indicates the AT content. The thick lines of the large circle indicated the extent of the inverted repeat regions (IRA and IRB) that separate the genome into small single-copy (SSC) and large single-copy (LSC) regions, respectively.

The complete cp genome of *B. flagellifera* was 125,025 bp in length (Figure 2). The overall AT and GC contents were 70.2% and 29.8%, respectively. The complete cp genome of this species has a typical quadrilateral structure consisting of large single-copy (LSC, 86,854 bp) and small single-copy (SSC, 18,473 bp) regions separated by a pair of 9849 bp-long inverted repeat (IR) regions. There were 127 genes in total: 82 protein-coding genes (PCGs), 37 tRNAs, and eight rRNAs (Figure S2). Of these, eight genes (*ndhB*, *ycf66*, *rpoC1*, *atpF*, *ycf3*, *clpP*, *rpl2*, and *ndhA*) are cis-spliced, and *rps12* is also a trans-spliced gene (Figure S3), similar to the gene arrangements of other Bryophyta cp genomes.

**Figure 3.**
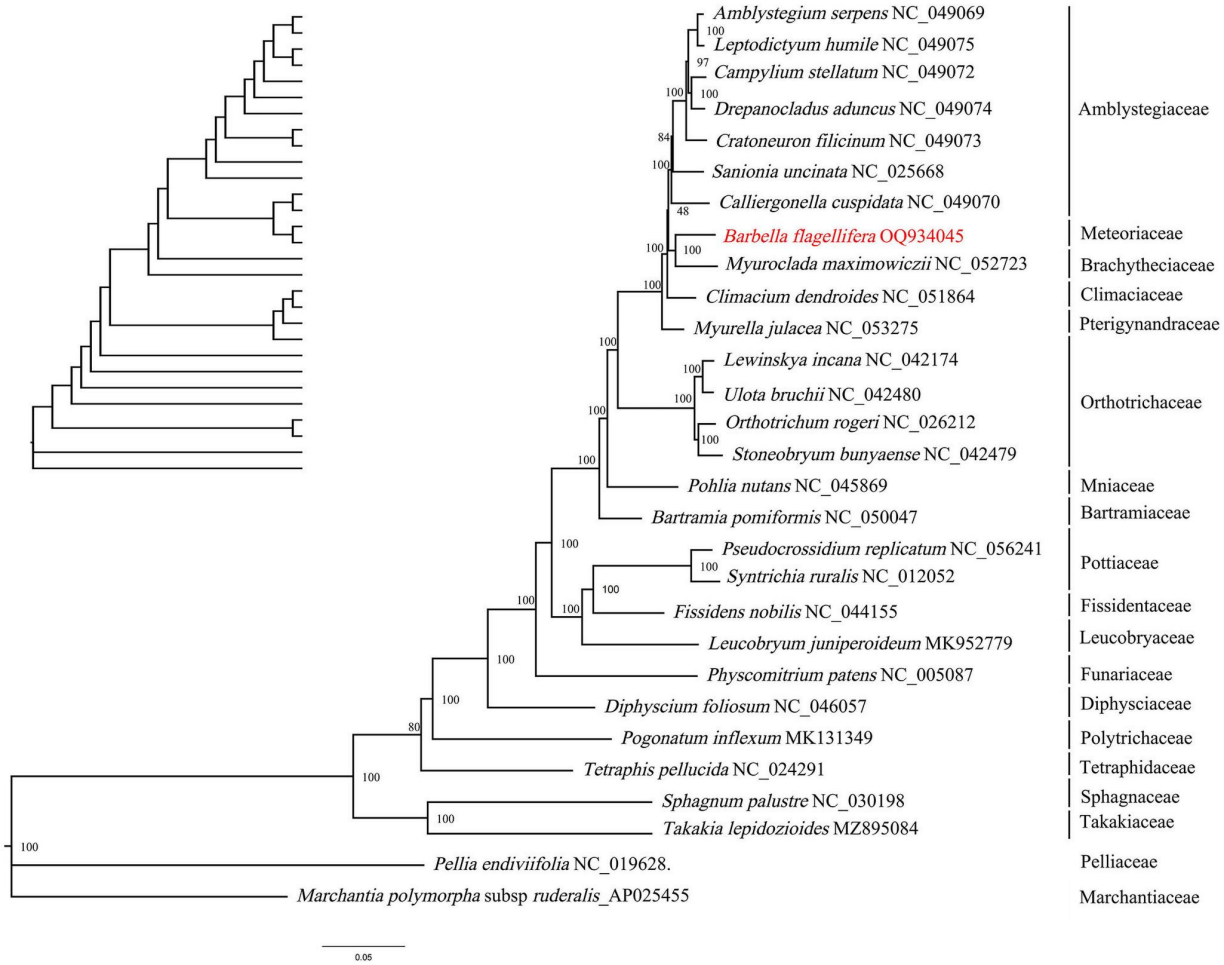
Phylogenetic relationships of *Barbella flagellifera* and other species of bryophyte inferred from ML analyses based on the whole chloroplast genome, with *M. polymorpha* (NC_037507) and *P. endiviifolia* (NC_019628) as the outgroups. The numbers on each branches indicate the boot support value of ML analyses. The sequences used for tree construction are as follows: *A. serpens* (NC_049069), *B. flagellifera*(NC_081510), *B. pomiformis* (NC_050047), *C. cuspidata* (NC_049070, Wei et al. 2020), *C. stellatum* (NC_049072, Han et al. 2020a), *C. filicinum* (NC_049073, Wei et al. 2020), *D. foliosum* (NC_046057), *D. aduncus* (NC_046057), *F. nobilis* (NC_046057, Kwon et al. 2019), *L. humile* (NC_049075, Wei et al. 2020), *L. juniperoideum* (MK952779, Min et al. 2019), *L. incana* (NC_042174), *M. maximowiczii* (MT726030, Han et al. 2020b), *O. rogeri* (NC_026212), *P. patens* (NC_005087), *P. inflexum* (MK131349), *P. nutans* (NC_045869, Jin et al. 2020), *P. replicatum* (NC_05624, Cevallos et al. 2019), *S. uncinata* (NC_025668, Park et al. 2018), *S. palustre* (NC_030198), *S. bunyaense* (NC_042479), *S. ruralis* (NC_012052), *T. lepidozioides* (MZ895084, Sadamitsu et al. 2021), *T. pellucida* (NC_024291, Bell et al. 2014), and *U. bruchii* (NC_042480).

A phylogenetic tree was constructed based on the complete cp genome of *B. flagellifera*, 27 complete cp genomes of Bryophyta, one Marchantiophyta genome and one Anthocerotophyta genome downloaded from NCBI; the cp genomes of *Marchantia paleacea* and *Pellia endiviifolia* functioned as outgroups. An ML tree was inferred based on the GTR + R4 + F matrix-based model using IQTREE2 with 1000 bootstrap replicates (Figure 3). The phylogenetic results indicated that *B. flagellifera and M. maximowiczii* formed a monophyletic clade with bootstrap support (100%). This suggests a close genetic relationship between the two species, although *B. flagellifera* belonged to Meteoriaceae, *M. maximowiczii* belonged to Brachytheciaceae. In addition, *B. flagellifera*, *M. maximowiczii*, and *C. dendroides*, three species of the pleurocarpous group are clustered together between the acrocarpous group.

## Conclusions

In this study, the complete cp genome of *B. flagellifera* was assembled for the first time and the structure of this species was annotated. It was found to have a typical circular form composed of genes of 125,025 bp. The phylogenetic analysis of *B. flagellifera* datasets provides convincing evidence that supports many traditionally recognized families. This suggests a close genetic relationship between *B. flagellifera* and *M. maximowiczii.* These findings would help to understand the genetic information; our research result provides basic genetic resources for the development of species identification of the *Barbella* genus and *Neodicladiella* genus in Meteoriaceae.

## Supplementary Material

Supplemental Material

Supplemental Material

Supplemental Material

## Data Availability

The genome sequence data that support the findings of this study are openly available in GenBank of NCBI at https://www.ncbi.nlm.nih.gov/ under the accession OQ934045. The associated BioProject, SRA, and Bio-Sample numbers are PRJNA921716, SRR23095850, and SAMN32630385, respectively.
