# Supplementary material for: Characterization of the complete chloroplast genome of Barbella flagellifera (Cardot) Nog. 1938 (Bryidae, Meteoriaceae)
Source: Mitochondrial DNA B Resour. 2024 Feb 26;9(2):304–8. doi: 10.1080/23802359.2024.2318393 (PMC10898263; doi:10.1080/23802359.2024.2318393)
Supplement: Supplemental Material [file TMDN_A_2318393_SM9185.pdf]

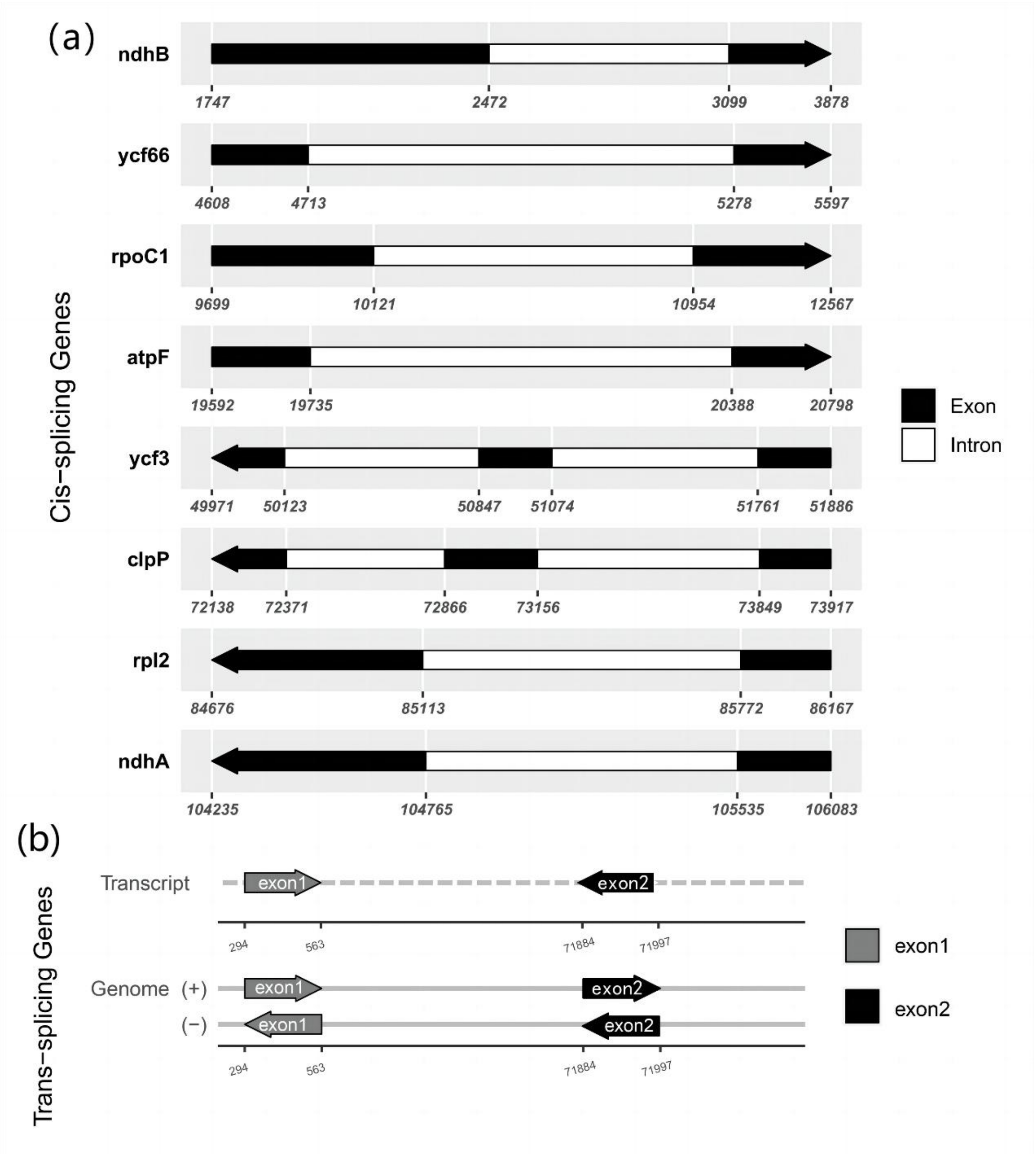

Supplementary Figure S3. Schemstic map of the cis-splicing and trans-splicing genes in the chloroplast genome of *Barbella flagellifera.* (a)The genes are arranged from top to bottom based on their order in the chloroplast genome. The gene names are shown on the left, and the gene structures are on the right. The exons are shown in black; the introns are shown in white. The arrow indicates the sense direction of the gene. (b) The trans-spliced gene *rps12* has two unique exons.
