# Supplementary material for: Characterization of the complete chloroplast genome of Barbella flagellifera (Cardot) Nog. 1938 (Bryidae, Meteoriaceae)
Source: Mitochondrial DNA B Resour. 2024 Feb 26;9(2):304–8. doi: 10.1080/23802359.2024.2318393 (PMC10898263; doi:10.1080/23802359.2024.2318393)
Supplement: Supplemental Material [file TMDN_A_2318393_SM9178.pdf]

(a)

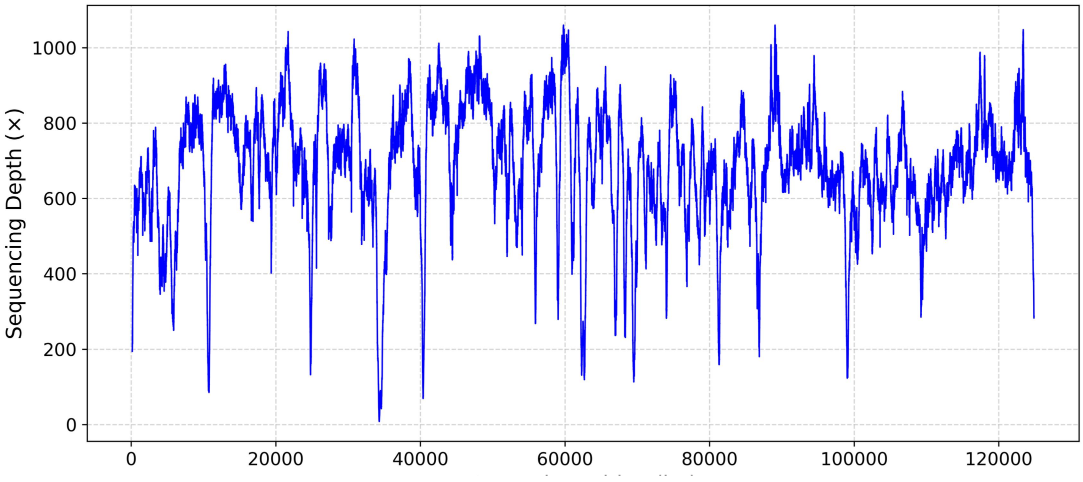

(b)

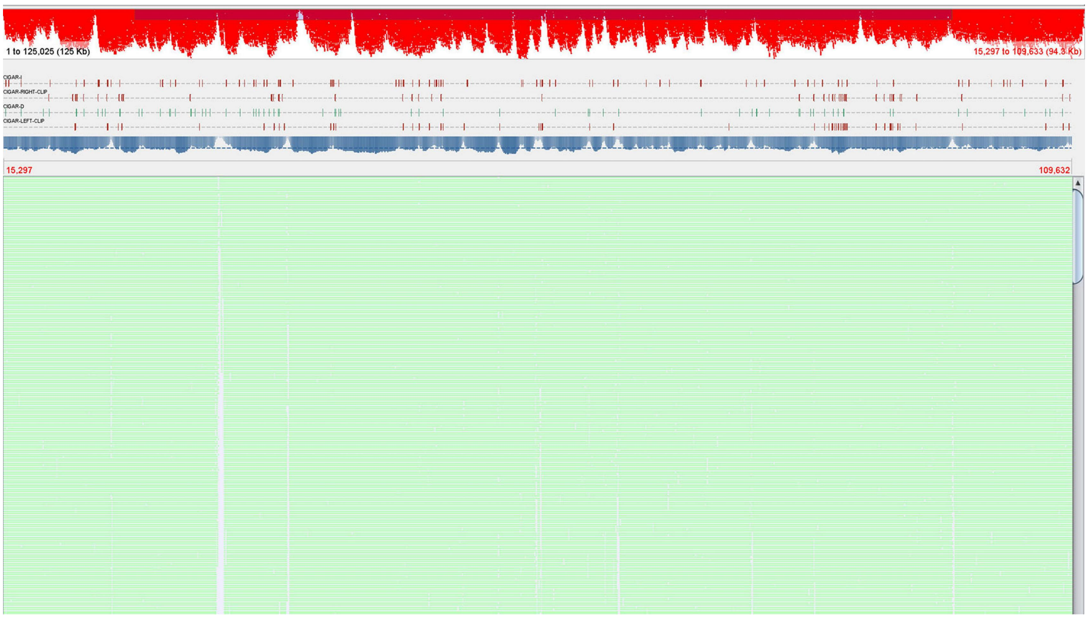

Supplementary Figure S1. Sequencing Depth and Coverage Map . (a) Clean read mapping depth is presented with blue bars. X and Y axis present genomic position of plastome and sequencing depth, respectively. The maximal depth is ×1060, the minimal depth is ×8, average depth is ×685.66.(b)The sequence coverage of the reference genome is presented with red bars. A gradient blue is used to show the depth of coverage of reads in the reference sequence.The green part shows the 150 bp fragment generated by sequencing.
