# Supplementary material for: Characterization of the complete chloroplast genome of Barbella flagellifera (Cardot) Nog. 1938 (Bryidae, Meteoriaceae)
Source: Mitochondrial DNA B Resour. 2024 Feb 26;9(2):304–8. doi: 10.1080/23802359.2024.2318393 (PMC10898263; doi:10.1080/23802359.2024.2318393)
Supplement: Supplemental Material [file TMDN_A_2318393_SM9183.pdf]

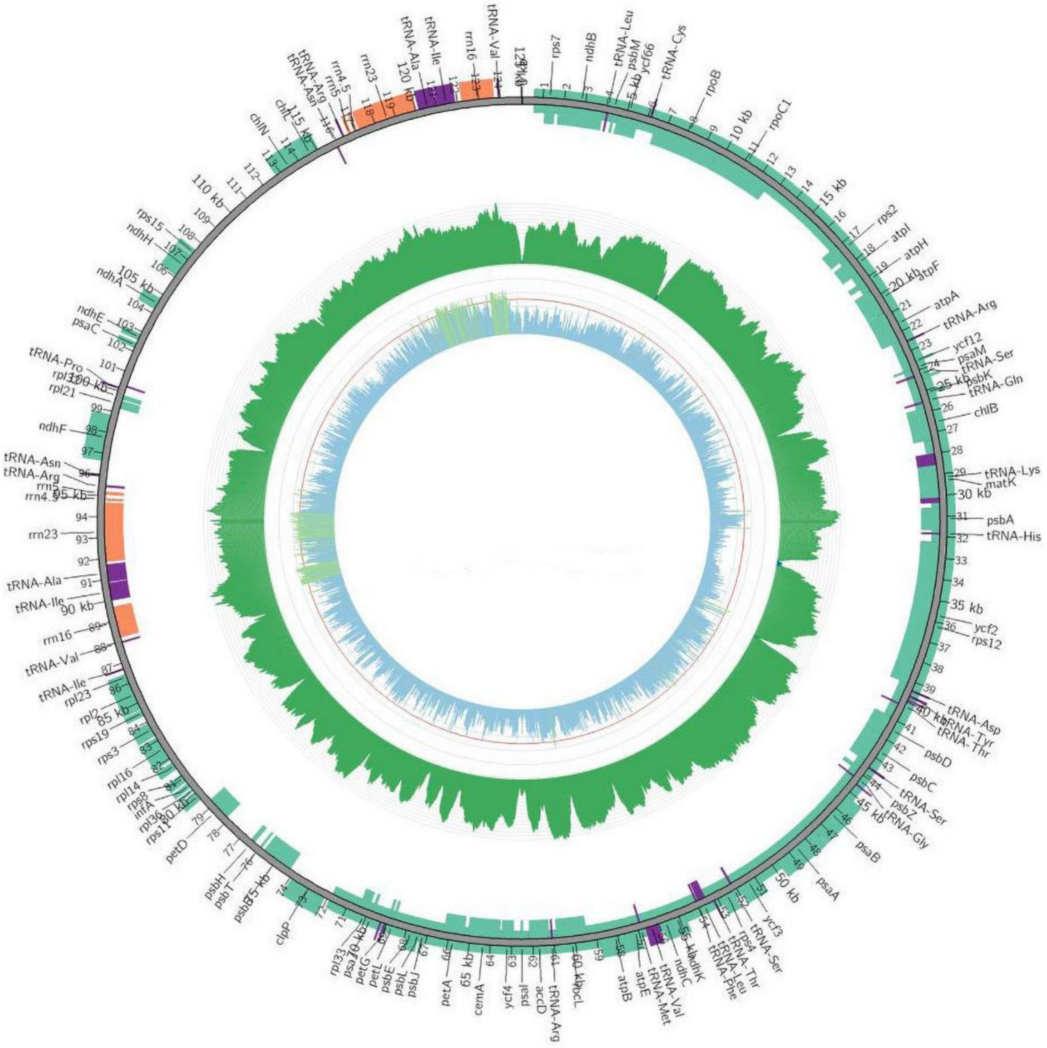

Supplementary Figure S2 Genome map of the assembly sequence. The outermost circle represents the genome sequence; the coding gene is represented by a green box; the t RNA is represented by a purple box; the rRNA is represented by an orange box; the green bars inside represent the depth of coverage; the innermost circle represent the GC content of the genome and the regions with more than 50% GC content are represented by green lines ,and vice versa by blue lines.
